# Refining the *Galleria mellonella* Model by Using Stress Marker Genes to Assess *Clostridioides difficile* Infection and Recuperation during Phage Therapy

**DOI:** 10.3390/microorganisms8091306

**Published:** 2020-08-27

**Authors:** Janet Y. Nale, Mahananda Chutia, Jeffrey K. J. Cheng, Martha R. J. Clokie

**Affiliations:** 1 Department of Genetics and Genome Biology, University of Leicester, Leicester LE1 7RH, UK; jn142@le.ac.uk (J.Y.N.); J.Cheng.7@warwick.ac.uk (J.K.J.C.); 2 Pathology and Microbiology Division, Central Muga Eri Research & Training Institute, Assam 785700, India; mahanaba@yahoo.co.uk

**Keywords:** *Clostridioides difficile*, *Clostridium difficile*, *Clostridiodes difficile* infection, *Galleria mellonella*, bacteriophage therapy, qPCR

## Abstract

The *Galleria mellonella* is an effective model for probing *Clostridioides difficile* interactions with phages. Despite valuable insights from this model, the larvae are not easily amenable to assessing detailed clinical responses to either bacteria or phages. Here, larval survival, colonisation and toxin levels were compared to expression profiles of 17 *G. mellonella* stress genes to monitor *Clostridiodes difficile* infection (CDI), and recuperation during phage therapy. The larvae were infected with a ribotype 014/020 isolate and treated with an optimised phage cocktail. Larvae treated prophylactically with phages and the phage-control larval group were protected, showing the highest survival, and low *C. difficile* colonisation and toxin rates, compared to co-infection, remedial and bacterial-control larval groups. Expression of growth (9) and reproduction (2) genes were enhanced within prophylaxis and phage-control larval groups compared to the co-infection, remedial and bacterial control groups. In contrast, expression of infection (2), humoral (1) and cellular (3) immunity genes declined in the prophylactic and phage-control groups but increased in the co-infection, remedial and bacterial control larvae. The molecular markers augment the survival, colonisation and toxin data and allow detailed monitoring of CDI and recovery. This data support the use of stress marker genes as tools to analyse clinical symptoms in this model.

## 1. Introduction

*Clostridioides difficile* (formerly, *Clostridium difficile*) is a toxin-producing bacillus and a major cause of antibiotic-induced diarrhea [1,2]. *C. difficile* infection (CDI) is characterised by one or more episodes of loose diarrhea, a positive stool test for *C. difficile* toxins A/B, and endoscopic evidence of pseudomembranous colitis, with or without a positive culture assay [3,4]. Other symptoms of CDI include moderate to severe lower abdominal pain, fever, anorexia, nausea, malaise and the presence of fecal occult blood. CDI may become life-threatening due to the progression to a toxic megacolon and death occurs in as many as 45% of cases [5,6,7]. The infection is difficult to treat, partly due to the protective spores and the biofilm the bacteria produce, that make the cells impenetrable to antibiotics [6,8,9,10]. Other reasons for the observed high mortality and morbidity may be attributed to the emergence and spread of antibiotic-resistant strains for which there are limited treatment options [11,12]. Currently, only three antibiotics (metronidazole, vancomycin and fidaxomicin) are approved for the treatment of CDI, but evidence for reduced susceptibility and resistance to metronidazole and vancomycin, respectively, have been reported [12,13,14,15]. Furthermore, the prohibitive cost of fidaxomicin limits its therapeutic use, particularly to strain-specific CDIs [16]. Generally, antibiotics kill other commensal bacteria in the gut leading to dysbiosis, which allows *C. difficile* to thrive, colonise and cause disease. Although new antibiotics and treatments such as fecal transplant are being developed, novel anti-infectives with target specificity and minimal deleterious impact on human gut microbial niche and immune system would be most appropriate to treat CDI [17,18].

Bacteriophages (phages) are viruses that target and kill bacteria. This selective phage–bacterial interaction can be harnessed for therapeutic purposes, and phage preparations for various human infections have been developed, and are alreadily available in some parts of the world [19,20,21]. In addition to specificity, phages replicate and accumulate at infection sites, hence their effective dose increases during therapy [20,22]. Although several well-characterised *C. difficile* phages have been reported in the literature, none have been developed for the treatment of the infection due to the lack of naturally occurring strictly lytic phages (lacking genes for lysogeny control) that target this pathogen [23]. Hence, to curtail lysogeny and resistance effects, we previously developed a novel phage cocktail containing four broad host range myoviruses [24]. The cocktail was shown to completely eradicate cultures of a clinically prevalent ribotype 014/020 and prevent the formation as well as disruption of established biofilms in vitro [24,25]. In addition to in vitro assays, the cocktail showed great efficacy at reducing *C. difficile* abundance in a batch fermentation model and favored proliferation of certain beneficial gut microbiota [26]. Moreover, a reduction in bacterial colonisation was also observed in the *Galleria mellonella* and hamster CDI models following treatment with the phage cocktail [24,25].

The hamster is a well-established in vivo CDI model because it demonstrates the most classical human CDI symptoms mentioned above [24,27]. Thus, the therapeutic potential of the optimised phage cocktail was easily extrapolated by linking animal survival and C. *difficile* colonisation rates with typical clinical manifestations demonstrated by the animals during infection and recuperation [24]. The *G. mellonella* larva model is particularly useful to circumvent ethical and cost issues relating to animal usage, and has previously been used to study the pathogenesis and virulence of various pathogens and other anti-infectives [28,29]. However, our previous report on CDI phage therapy using this model relied solely on C. *difficile* colonisation and larval survival data, which represent the end-stage of the disease [25]. Although visual inspection of changes in motility behavior and morphological features of the insects were helpful signs to ascertain when they were diseased and dead, these data are not easy to parametrize and quantify [25,30,31]. Other typical symptoms of the infection are also difficult to examine, due to the small size of the insects [25]. Although expressions of *G. mellonella* genes have previously been described as potential genetic tools to study the pathogenesis of bacteria such as *Listeria monocytogenes* and *Clostridium perfringens*, none have been described for CDI [28,29,32]. To address this limitation of the *G. mellonella* larva CDI model, this study was designed to establish the insects’ survival rates and levels of *C. difficile* toxins A/B during colonisation and different phage therapy regimens. The infection parameters were then related with the expressions of 17 previously reported *G. mellonella* stress marker genes relating to growth (9), infection (2) and reproduction (2), and humoral (1) and cellular (3) immunities within the experimental insects [28,29].

## 2. Materials and Methods

### 2.1. Bacterial Isolates and Inocula Preparation 

In this study, two *C. difficile* isolates were examined. The first, CD105HE1 is an environmental isolate of ribotype 076, and was used as a bacterial host for the propagation of the phages used here [33]. The second isolate, CD105LC2, was used for the in vivo infections, and is ribotype 014/020 [25]. The bacterial isolates were routinely cultured anaerobically (10% H_2_, 5% CO_2_ and 85% N_2_, MiniMACS Anaerobic Workstation, Don Whitley Scientific, West Yorkshire, UK) on brain heart infusion (BHI) agar (Oxoid, Basingstoke, UK) supplemented with 7% defibrinated horse blood (E & O Laboratories, Limited, Bonnybridge, Scotland) at 37 °C for 48 h prior to use. Cultures were stored in Viabank cryogenic storage tubes (Abtek Biologicals Ltd., Liverpool, UK) at −80 °C. The bacterial culture used for the larval infections was produced by inoculating a single colony of the test bacterium in 5 mL of pre-reduced BHI broth, and further incubating as above for 18–24 h. All liquid culture media were pre-reduced anaerobically at 37 °C for at least 1 h prior to use. A 10% volume of the overnight culture was prepared in fresh BHI broth and incubated until OD_550_ ~0.2 (~10^7^CFU/mL) was attained. The culture was centrifuged at 5000× *g* for 5 min and the pellet was re-suspended in an equal volume of ice-cold BHI. The centrifugation step was repeated two additional times to eliminate possible toxins that were released during bacterial growth. The final bacterial pellet was re-suspended in the ice-cold medium to give a final concentration of ~10^7^ CFU/mL, kept at the 4 °C and used within 2 h.

### 2.2. Preparation of Phage Cocktail

The four *C. difficile* myoviruses, CDHM1, 2, 5 and 6 used for phage therapy in this study were isolated and characterised previously in our laboratory [24,25,26,33]. The phages were propagated individually in liquid cultures of the environmental isolate, CD105HE1 to produce 10^10^ PFU/mL of infective phage particles [24,25]. Prior to use, the phages were diluted to 10^8^ PFU/mL in BHI and mixed in equal proportions to constitute the cocktail. The phage lysates were kept at 4 °C for short-term storage or in 25% glycerol for long-term storage at −80 °C.

### 2.3. Preparation of G mellonella Larvae and Model Set Up

*G. mellonella* larvae were obtained, prepared and set-up using optimisation procedures previously described [25]. Three sets of four larvae were used for each of the time points for the eight experimental treatment groups shown in Table 1. The first set of insects was used to determine the survival rate, and to conduct bacterial colonisation and phage recovery assays; the second set was used to assess toxin level and the third set was used for RNA extraction and qPCR analysis. The experiment was set up for 72 h and repeated three times.

To ensure that the stress levels exerted on the insects were as similar as possible, each insect in the eight treatment groups (except those in group 1) were treated twice only, with either an empty needle, 10 µL of either BHI, 10^5^ CFU of bacterial inoculum, 10^6^ PFU of phage cocktail and/or a co-infection of the bacteria and phage (Table 1). Larvae were transferred to sterile Petri dishes and kept at 4 °C until the first treatment (0), afterwards transferred to 37 °C before the second treatment (2 h), and further incubated at this condition for the rest of the duration of the experiment (up to 72 h).

### 2.4. Bacterial Infection and Phage Therapy Regimens in G. mellonella Larvae

The first treatment group consisted of the untreated insects. In this group, larvae were removed from 4 °C and incubated at 37 °C for the 72-h experimental period (Table 1). The second treatment group included larvae treated with empty syringes at the first and second time points to ascertain if any stress was inflicted on the insects from treatment with the needles during the experiment. The insects in the third group were treated at both time points (0 and 2 h) with cold BHI, which is the suspension medium for both the bacterium and phages. Procedures for treatments in the fourth (Control 4-Bacteria (CD105LC2), fifth (Phage/bacteria co-infection), sixth (Remedial regimen), seventh (Prophylactic regimen) and eighth (Control 5-Phage only) were detailed in Table 1 and in our previous publication [25].

### 2.5. Survival Rates, Bacterial Enumeration and Toxin Assay from the Treated Insects

Survival rates were recorded for all sets of insects at each time point (0, 2, 24, 48 and 72 h) in all the treatment groups. The scoring for dead/live and recovery of bacteria and phage from each of the larvae were carried out as previously described [25]. The toxin assay was conducted on extracted guts (into 1 mL PBS) of the second set of larvae for all the time points with *C. difficile* Tox A/B II™ (Techlab, Virginia, USA) in accordance with the manufacturer’s manual. Data were subjected to the Anderson–Darling and Shapiro–Wilk normality test (dependent on the sample size) to determine the applicable downstream data analysis. For two different treatment groups, the parametric test of choice was the two-tailed Students *t*-test and the non-parametric test was the Mann–Whitney–Wilcoxon test. Log Rank Mantel–Cox tests were conducted as the Kaplan–Meier survival curve analysis. Significance was denoted by asterisks, with * = *p < 0.05*, ** = *p < 0.01*, *** = *p < 0.001*, **** = *p < 0.0001* and n.s. = not significant.

### 2.6. RNA Extraction, cDNA Synthesis and qPCR

The third set of insects from each of the treatment groups were harvested for RNA extraction. Briefly, the insects from each time points were removed from 37 °C, placed in Falcon tubes and dipped in liquid nitrogen for 20 s and immediately transferred to −80 °C. RNA was extracted from the larvae using Trizol. Briefly, one randomly selected previously live (before dipping in nitrogen) larva from each treatment group was transferred into ice-cold 2 mL capacity chilled (for at least 15 min on ice) screw-cap micro tubes (Sarstedt, Nümbrecht Germany) containing 500 mg of 106 µm size acid-washed glass beads (Sigma-Adrich, Missouri, USA) and 900 µL Trizol (Ambion, Life Technologies, Carlifornia, USA), 200 µL each of RNAprotect Tissue Reagent (Qiagen, Manchester, UK) and chloroform. The tubes were further incubated at room temperature for 5 min, and then homogenised using PowerLyzer 24—Bench Top Bead-Based Homogenizer (Cambio Ltd., Dry Drayton, UK) instrument at setting 6.0 m/s for 40 s. The tubes were removed and incubated at room temperature for a further 5 min time followed by centrifugation at 12,000× *g* for 15 min. The upper phase containing the RNA was extracted and the RNA quality and quantity was determined using NanoDrop One (Thermo Scientific, Madison, USA). The RNA samples were aliquoted into sterile ice-cold microtubes and immediately stored at −80 °C. For experimental group 4, insects for timepoint 72 h were examined at ~65 h to ensure live insects were used.

cDNA was synthesised from ~500 ng of the RNA (in 1 µL) using the RevertAid First Strand cDNA Synthesis Kit with random hexamer primers according to the manufacturer’s instructions (Thermo Fisher Scientific, Vilnius, Lithuania). Approximately, 50 ng of the synthesized cDNA was subjected to qPCR using the 7500 Fast Real Time PCR system with Fast SYBR Green Master mix (Applied Biosystems, California, USA) and 125 µM primers targeting the previously published 17 *G. mellonella* stress markers (Table 2) [28]. Data was normalised with *G. mellonella* housekeeping 18S RNA genes and analysed using 7500 Fast SDS software v2.3 and GraphPad Prism 8.

## 3. Results

In this study, we aimed to determine the responses of *G. mellonella* larvae to CDI and recuperation to different phage therapy regimens. To do this, we first infected the larvae with cultures of a clinically prevalent human isolate (CD105LC2, ribotype 014/020) and determined the rates of *C. difficile* colonisation and toxin production within the insects, as well as recording survival rates at the 0, 2, 24, 48 and 72 h time points. These parameters were then correlated to the expressions of 17 previously reported *G. mellonella* stress-related genes during all the treatments as quantified by qPCR [28].

### 3.1. Impact of Phage Therapy Regimens on G. mellonella Survival 

**Figure 1:**
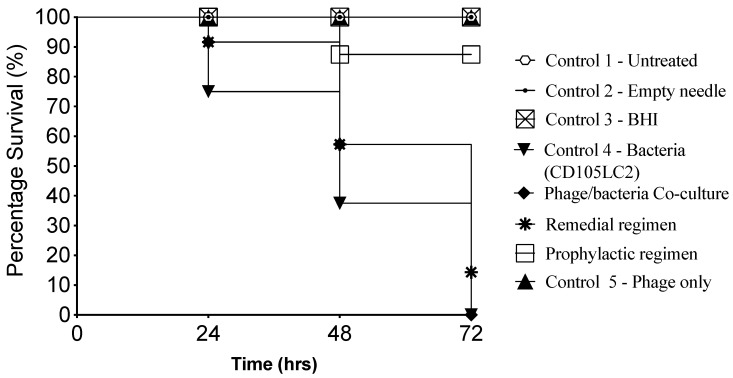
Kaplan–Meier survival curve of *G. mellonella* infected with CD105LC2, a clinically relevant ribotype 014/020 isolate, and treated with a 4-phage cocktail in 3 therapy regimens. The insects were treated prophylactically or remedially with the phage cocktail, or with a co-infection of the bacteria and phages. The treatment regimens were compared to Controls 1–5 for untreated insects, or those treated with empty needles, BHI, bacteria or phage cocktail, respectively, as shown in Figure 1 and Table 1. The prophylactic regimen performed best when compared with the remedial regimen (*p* < 0.01) and co-infection group (*p* < 0.01). Four larvae were examined per treatment group and the experiment was repeated on three occasions. Data was analysed using the Log Rank Mantel–Cox test in GraphPad Prism 8.

Among the three therapy regimens investigated here, as expected, insects in the prophylaxis group were best protected from the infection, thus, a 100% survival rate was recorded throughout the experiment, which is significant when compared to the bacterial control (*p* < 0.001). Among the insects treated with a co-infection of the phage cocktail and bacteria, the first fatality was recorded in the first 24th hour, when ~10% of the insects died. As the experiment progressed, the co-infection group exhibited a similar reduction in survival rates to the bacterial control group. The least efficient treatment regimen was observed in the remedial group, where over 40% of the insects died in the first 48th hour post-infection, and fatality reached ~85% at the 72nd hour. Insects in all the control groups survived except those in the bacterial control group, where 10%, 40% and 100% of the larvae died at the 24th, 48th and 72nd hour, respectively, post-infection (Figure 1).

### 3.2. Impact of Phage Therapy Regimens on C. difficile Colonisation on G. mellonella

**Figure 2:**
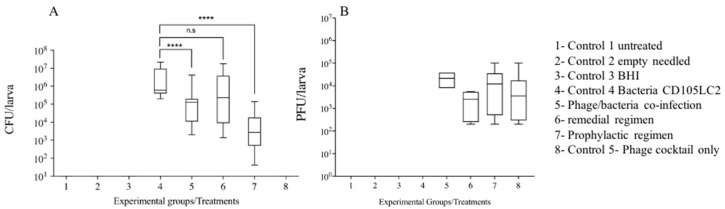
Bacterial and phage load recovered from *G. mellonella* larvae at the end of the 72nd hour. Four larvae in each of the eight treatment groups were treated with 10^5^CFU/larva of clinical isolate CD105LC2 culture and/or 10^6^ PFU/larva of phage cocktail (in 10 µL volumes) at time points shown in Table 1. Colonisation was ascertained by recovering (**A**) *C. difficile* from the guts of insects on CCEY selective plates and (**B**) phages using double BHI agar method. The lowest amount of bacteria was recovered from the prophylactic treatment (compared to the bacterial control), indicative of successful phage infection (*p <* 0.0001). The co-infection (with phage and bacteria) treatment also yielded a reduced bacterial load (*p* < 0.0001). The experiment was repeated three times and data were analysed using the Shapiro–Wilk normality test and Mann–Whitney–Wilcoxon test. n.s. = no significant difference, **** = significance at *p* < 0.0001.

At the end of the treatments (72 h), bacterial colonisation rates within the larvae were ascertained by recovering *C. difficile* from the gut contents of individual insects on CCEY selective medium and incubation under anaerobic conditions [25]. Bacteria were undetected in experimental groups 1 (untreated), 2 (empty needles), 3 (BHI), and 8 (phage control) (Figure 1) during the various time points as these groups of insects were not treated with bacteria at the beginning of the experiment. In contrast, within the bacterial control group (control 4), as expected, colonisation progressed successfully in the insects from the initial inoculum of ~10^5^ CFU/larva administered to ~10^6^ CFU/larva recovered at the final time point (72nd hour). Comparing all phage treatment groups, a log-fold CFU/larva reduction in bacterial counts was observed within the insects treated with a co-infection of the phages and bacteria (experimental group 5). In the remedial regimen group (experimental group 6), no log-fold CFU/larva reduction was observed at the end of the experiment. As expected, the greatest bacterial load reduction (~2 log CFU/larva reduction) was observed in the prophylactic group (experimental group 7), and only ~10^4^ CFU/larval of *C. difficile* were recovered at the end of the experiment (Figure 2A). Resultant phage recovery at this time point showed higher numbers ~10^5^ PFU/larvae in treatment groups 5, 7 and 8, and 1 log lower in the treatment group 6, which is the remedial regimen (Figure 2B).

### 3.3. Impact of Phage Therapy on C. difficile Toxins Levels in G. mellonella

To further correlate bacterial load within the insects as colonisation progressed during infection or declined during phage therapy regimens, we measured the accumulation of *C. difficile* toxins in the haemolymph of the insects at each time point. The ELISA-based toxin assay utilises highly specific antibodies to detect either Toxin A or B within the sample and quantifies the relative expression to a proportional OD_450_ value. The minimum threshold for a positive sample is an OD_450_ value greater than 0.120; a strong positive for a *C. difficile* toxin is represented by any OD_450_ value above 0.200. As expected, we observed that the levels of toxins were low and hence barely detectable among the negative control groups 1, 2, 3 and 8, which correlate to the untreated, needle-treated, BHI and the phage-only treated groups, respectively (Table 3). Among the bacteria-only treated insects (group 4), however, toxin production progressed steadily by the observation of the rise in OD_450_ values of the toxins at the experimental time points (OD_450_ of 0.072 at 2 h to 0.234 at the 72 h), which also concurred with the increase in colonisation within the insects (Figure 2). Observation of toxin levels in the phage remedial group (group 6) showed increased toxins (from OD_450_ 0.108 to 0.293) after 24 h. This might be attributed to bacterial lysis due to phage infection leading to toxin accumulation in the guts of the insects. After this time, toxin levels tended to fluctuate from 0.223 at 48 h to 0.260 at the end of the experiment. The toxin levels were consistently lower in the co-infection and prophylaxis groups (groups 5 and 7, respectively) compared to the remedial group at all the time points (Table 3). The prophylactic group displayed an elevated concentration of toxin levels within the first 24 h, before it declined to below the minimum threshold at 48 h. The toxin data strongly correlates with the amount of bacterial load recovered in Figure 2. Although at 72 h there was a slight increase in OD_450_ value, this was within the borderline toxin-positive value.

Hemolymph and extracted guts of uninfected and infected larvae were subjected to a toxin assay at each respective time point. Samples were read in a POLARstar Omega plate reader (BMG LabTech, Germany) at OD_450_. Weak positive samples had an OD_450_ value of 0.120 to 0.199, strong positive samples displayed an OD_450_ value of 0.200 and above. The efficacy of phage therapy was most dominant in the prophylactic regimen, similarly observed in the bacterial recovery of infected larvae. A reduction in toxin expression was also observed in the co-infection treatment at 72 h.

### 3.4. Gene Regulation during Infection and Phage Therapy in G. mellonella

Having established the survival and colonisation rates of the insects during the course of CDI and phage therapy, we extracted total RNA from the insects at the time points (0, 2, 24, 48 and 72 h) and determined their relative stress response to the infection and phage therapy. The responses were ascertained by determining the expression levels of 17 *G. mellonella* stress-related genes using RT-qPCR and comparing the data with the larval survival, and *C. difficile* colonisation and toxin A/B production rates at each time point. The genes examined were related to growth (9), humoral (1) and cellular immunity (3), infection (2) and reproduction (2) (Table 2).

#### 3.4.1. Impact on Growth Markers

**Figure 3:**
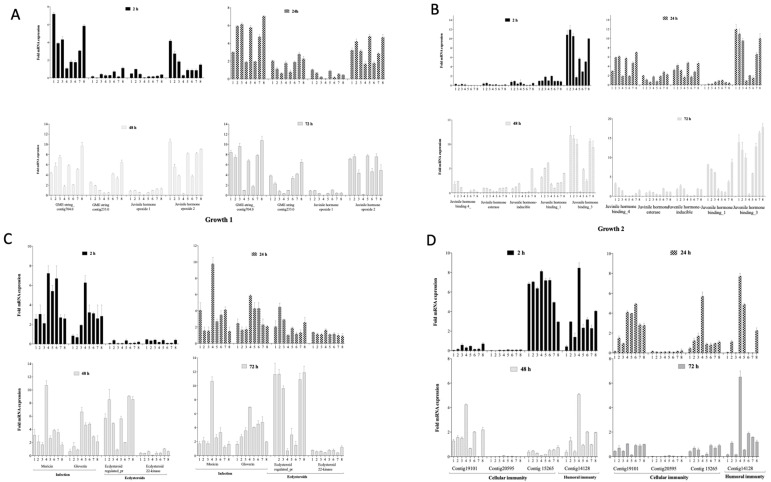
Expression of 17 *G. mellonella* growth markers during *C. difficile* colonisation and phage therapy regimens. The relative expression levels of (**A**, **B**) 9 growth, (**C**) infection and reproduction and (**D**) cellular and humoral genes were examined during *C. difficile* colonisation in *G. mellonella* larvae and when undergoing different phage therapy regimens. *G. mellonella* larvae were either untreated (1), treated with empty needles (2), 10 µL of either BHI (3), or 10^5^ CFU/mL of CD105LC2 bacterial culture (4). The larvae were also treated with a co-infection of the phages (10^6^ PFU/mL) and bacteria (5), remedially (6) or prophylactically (7), and compared to a group of larvae treated with just the phage cocktail (8). Experiments were repeated three times. Values were normalised using the housekeeping gene 18S rRNA and analysed using GraphPad Prism 8.

When the nine growth related markers were examined, it was observed that the Juvenile hormone epoxide hydrolase_1 gene was not affected by any of the treatments at all the time points. The gene was expressed at very low levels (<2-fold mRNA expression) in the control groups 1 (untreated), 2 (empty needle), 3 (BHI), 4 (Bacteria) and 8 (phage only) during all the experimental time points. Similarly, low expression levels of this gene at these times were also observed in the larval co-infection (treatment 5), remedial (treatment 6) and prophylactic (treatment 7) treatment groups (Figure 3A).

In contrast, three genes: GME-string_contig704.0, Juvenile hormone epoxide hydrolase_2 and Juvenile hormone binding_3 were highly expressed (4–12 mRNA fold change) in the control groups 1, 2, 3 and 8. However, it was observed that the three genes were least expressed (~2-fold mRNA fold change) in the bacteria-only treated group (group 4). Considering the three phage treatment regimens, insects treated with a co-infection of the phage and bacteria simultaneously (group 5) and those treated prophylactically (group 7) had invariably greater mRNA expression levels (2–7 and 3–8 mRNA fold change, respectively) of these three growth genes. The expression of the genes in insects within the remedial group (group 6) revealed much lower mRNA fold change levels (1–4) compared to the other treatment regimens and controls 1, 2, 3 and 8, but much higher when compared to group 4 (Figure 3A,B).

The remaining five growth genes (GME-string_contig233.0, Juvenile hormone binding_4, Juvenile hormone esterase, Juvenile hormone inducible and Juvenile hormone binding_1) showed low level expressions (<2-fold mRNA expression) during the first 2 h in all the treatment groups. However, at 24 h, the five genes became eminently expressed (3–7-fold mRNA expression) in all the treatment groups. In the 48 and 72 h, the expression levels of three (Juvenile hormone binding_4, Juvenile hormone esterase and Juvenile hormone inducible) of the five genes dropped to <3-fold mRNA expression in both times points. An exception was observed in the prophylactic group for the Juvenile hormone inducible gene, where ~6-fold mRNA expression level was observed at 48 h in this treatment group.

#### 3.4.2. Impact on Markers of Infection, Molting and Reproduction

Having examined the impact of the bacterial infection and phage therapy regimens on the larval growth markers, we determined the impact of the treatments on two additional genes that relate to infection (moricin and gloverin), and two that are involved in reproduction and molting in the insects (Ecdysteroid-regulated protein and Ecdysteroid 22_kinase). Generally, all larvae undergoing phage therapy regimens (groups 5, 6 and 7) expressed the infection genes less than the bacteria-infected control (group 4) but more highly than the negative control 1, 2, and 3 groups (Figure 3C).

For the two infection genes examined, we observed that moricin was more highly expressed during the first 2 h of the experiment, having 3–8-fold mRNA expression, than the gloverin with 2–7-fold mRNA expression levels in the controls as well as the treatment groups at this time (Figure 3C).

During the first 2 h, the phage therapy regimens 5 (bacterial infected simultaneously with the phage) and 6 (remedial regimen) seemed to exhibit high expression levels (6–8-fold mRNA expression) of the moricin marker, and these are comparable to the bacterial control 4 having 7–8 mRNA expression level. At this time point (2 h) the prophylactic regimen (group 7) showed lower expression level of the gene, having ~3 mRNA expression at this time point. At 24 h, however, the expression level of the moricin gene increased from ~2 to ~5 in the untreated group (control 1) but later decreased to ~4 and ~2 on the 48 and 72 h, respectively, in the same group (Figure 3C). Similarly, in the control 2, 3 and 8 groups, the moricin gene expression dropped from 2–4 mRNA expression in the 24 h to <2 mRNA expression in the 48 h and stayed at this level until 72 h. From the 24 h to 72 h of the experiment, the larvae infected with the bacteria alone in the bacterial control 4 showed a significantly high level of this infection gene compared to the other treatment groups (Figure 3C).

Similarly in the gloverin, all the negative controls 1, 2 and 3 exhibited low levels (<1 m RNA expression) of the gene at the 72 h, and at the 24 h and 48 h too. At 72 h, however, these groups of insects expressed this gene to 2–4 mRNA expression level. When compared, the phage therapy regimen groups and the phage control group 8 expressed the gloverin gene more highly (3–4 mRNA expression) than the negative control groups 1, 2 and 3 (<1) at the 72 h. However, at the 24 h time, the prophylactic and the phage groups had ~2 mRNA expression, which is comparable to the negative control groups 1, 2 and 3. In contrast, the remedial group and larvae group treated with phage and bacterial culture showed relatively high expression levels of ~6 mRNA expression from 24 h to 72 h (Figure 3C).

Regarding the impact on the insects’ reproduction and molting, we examined the Ecdysteroid-regulated protein and Ecdysteroid 22_kinase. The latter showed very low expression levels throughout the experiment in all the groups (<2 mRNA expression). However, the Ecdysteroid-regulated protein expression was very low (<2 m mRNA expression) until 24 h when the fold mRNA expression reached 11-fold mRNA expression in the treatment groups. Specifically, the bacterial control and the remedial treatments were expressed at low levels (<2 mRNA expression), while the prophylaxis and the treatment with the phage and bacterial co-infection were highly expressed ranging between 6 and 10 mRNA expression and these levels are comparable to the untreated, and larvae treated with just empty needles, BHI and with phages.

#### 3.4.3. Impact on Cellular and Humoral Immunity Markers

The next group of stress markers examined were those associated with cellular and humoral immunity of the insects (Figure 3D). Among all the three genes related to cellular immunity, Contig19101_1 and Contig20595_1 were barely expressed at the first 2 h but Contig15265_1 was eminently expressed reaching up to 8-fold mRNA expression at this time point. At 24 h, however, the expression of Contig15265_1 genes became drastically reduced, just barely reaching 2-fold mRNA expression in all the treatment and control groups, although the untreated seemed to show lower expression level at this time point. Additionally, at 24 h, it was observed that expression levels of Contig19101_1 and Contig20595_1 genes were raised (reaching 4–5-fold mRNA expression levels) in all treatments and controls except in the untreated insects and those treated with empty needle and BHI. In the humoral response, high levels were generally observed in the pure BHI-treated groups. Although up to 4-fold levels were observed in the other groups within 2 h post phage treatment, the expression levels dropped in other groups except those in co-infection and phage-only groups.

## 4. Discussion

The dynamics of phage–bacterial interactions are highly complex [34]. Various models have been designed to explore aspects of these interactions and data provide useful insights into the potential applications of phages, whether as diagnostic tools or as therapeutic agents [20,24,25,26,35,36]. In vitro models are particularly useful as they allow detailed probing of various aspects of bacteria and phage activity, both in pure as well as in co-infection environments. In addition, the in vitro system has a greater flexibility of including multiple replicates, and controls to robustly inform animal and subsequent pre-clinical data for downstream phage applications. However, phage interactions with cells and immune systems are difficult to determine in vitro, even in cell tissue systems [36]. To address these difficulties here, we showed the application of the *G. mellonella* CDI model as a refined in vivo CDI phage therapy model and examined ways to improve this system.

The use of alternative in vivo models instead of complex larger models forms part of the 3Rs (replacement, reduction and refinement) guiding principles regulating animal use in scientific research [37]. Larger animal models are useful in their merit in that they are well established, and could display classical symptoms of diseases comparable to those found in humans [27]. Despite its clinical advantages, larger animal testing has great inherent limitations in research. The limitations are compounded by high costs relating to complex care and husbandry, long and tedious procedures for obtaining licenses, limited replicates and small population size to be examined at a time due to space [25,37]. Due to these fundamental flaws, researchers have now shifted interests towards the use of small invertebrates such as zebrafish (*Danio rerio)* embryos and wax moth (*G. mellonella*) larvae to study various aspects of bacterial pathogenicity and infection [28,29,38,39]. Wax moth larvae are, particularly of great importance in both infection and therapeutic intervention studies because they possess antimicrobial defences and complex innate immune systems. They also have phagocytosing cells in their haemolymph and possess epithelial-like cells in the mid-gut that are comparable to those found in humans. Hence, they are widely used in infection studies and can provide insight into the pathogenesis and virulence of human pathogens [28,32,39,40,41].

Pertinent to CDI phage therapy studies, mammalian models such as the hamster CDI infection model have been widely described for these purposes [24,42,43]. Whilst hamsters show classical human CDI symptoms, they are particularly sensitive to *C. difficile* toxins, hence they may lead to exaggerated fatal cases and survival data [44]. On the other hand, the non-classical wax moth larval CDI model can reveal the therapeutic potential of phages by the colonisation and survival data; however, the insects’ extremely small size (~250–300 mg, 2 cm long) meant that typical clinical symptoms of the disease are impossible to be ascertained [25,39]. Therefore, we reported here the expression of the insects’ stress marker proteins during CDI and phage therapy regimens to circumvent this inherent difficulty and assess infection and recuperation processes within the insects in greater detail.

The phage therapy regimens examined here were conducted using a 4-phage cocktail, which was previously optimised for the effective eradication of the *C. difficile* CD105LC2 (ribotype 014/020) cultures in vitro, to prevent the establishment and proliferation of the bacterium in biofilms, and to reduce colonisation in both hamster and *Galleria* CDI models [24,25,26]. The phages in the mix have complementary properties, in that one phage-resistant/lysogenic strain produced from infection from one phage is susceptible to infection by a different phage in the mix [21]. In our previous phage therapy studies using the *G. mellonella* CDI model, we clearly demonstrated the colonisation profiles and the survival rates of insects during therapy regimens with the 4-phage cocktail examined in this study [25]. In order to examine the physiological conditions of the larvae during CDI infection and phage therapy regimes, it was essential to repeat the therapy assays. However, in addition to the survival and colonisation data, we not only examined the *C. difficile* toxins A/B accumulation during infection and recuperation during phage therapy, but also determined the expression of the insect stress genes as indicators of the disease. Our current observations on the *C. difficile* colonisation and insects’ survival rates in the controls and phage therapy regimens concur strongly with our previous findings [25]. This further strengthens the robustness of the model for CDI and phage therapy studies. However, because our current study entailed the determination of gene expressions, the experimental set-up was re-designed to include three additional controls; insects treated with just brain heart infusion (BHI) medium and empty needles, and the untreated group. This was to enable the probing of different potential factors that may trigger any stimulus in the insects and interfere with the expression of the various genes examined.

Although BHI contained sources from animal origin, we did not observe any detrimental physical reactions on insects within the BHI treatment group medium as shown previously. Therefore, all insects within this treatment group survived as previously reported. We also observed complete survival in the untreated and needle-only groups [25]. This suggests that the oral gavage method is effective at delivering the samples to the guts of the insects without subjecting them to unnecessary adverse physical stress, as shown previously [25,39,45,46]. In addition to survival, the insects within these groups tended to express all the growth genes at high levels except at 24, 48 and 72 h of contig_704.0 gene and 24 h of Juvenile hormone binding 4, where the BHI-treated groups expressed the genes more at these time points. High expression was also observed on the Ecdysteroid-regulated_protein gene responsible for reproduction. This observation may be attributed to the glucose content of the BHI medium, which was shown to act as a feeding stimulant and permit optimum growth and development in insects [47]. Further studies on the role of glucose in the development of insects was demonstrated in the brown planthopper. Studies showed that silencing the glucose transporter gene 6 *Nlst6*, resulted in prolonging the pre-oviposition period, shortening the oviposition period and reducing the body weight of the brown planthopper [48]. Although the insects in our study were not fed during the experiments, glucose present in the BHI not only provided nutrition but could enhance reproduction within the insects.

Of the three cellular immunity genes examined, two were expressed and one was not activated, as seen previously in work conducted with *L. monocytogenes* [49]. Previous work analysing the proteome of starved, incubated and food-deficient larvae indicated a decrease in the abundance of a range of proteins associated with the immune response [50]. Since insects were not fed during the experimental time; this might elucidate the diverse patterns of responses we observed among the cellular as well as the humoral immunity genes, howbeit, further work is needed to determine these differences.

The hemocytes found in the digestive tract of the insects modulate cellular response during infection, leading to the phagocytosing and neutralisation of pathogens by the production of superoxide and lytic enzymes [51]. Specifically, the cellular immunity gene, Contig19101_1, responsible for the function of neuromuscular junction development, regulation of cell shape, cortical actin cytoskeleton organisation, phagocytosis, engulfment, axonogenesis, oocyte growth, cell projection assembly and myoblast fusion, was slightly increased after 2–48 h (2–4-fold) in the bacterial control [28]. Similarly, Contig20595_1, which is involved with the G-protein signalling pathway and response to stress, was expressed only after 24 h of post infection in the remedial and prophylactic larval groups [28]. The timing of the expressions of these genes (at 24 and 48 h) concurs with previous observation that pre-incubation of the larvae at 4 °C or 37 °C exposed them to mild thermal shock, leading to an increase in hemocyte density and peak expression of immunity-related genes within 24 h post infection with *Candida albicans* [52]. The expression of the genes for an extended 48 h in this study might be attributed to the fact that larvae used here were kept at 4 °C upon arrival, without food for up to a week before usage, and this might have affected the abundance of immune-related proteins seen here.

We also observed fluctuations in the humoral immunity genes, but the genes were consistently high in the bacterial-only treated control group. The genes are associated with the defence response to Gram-positive bacterium, innate immune response, xenobiotic metabolic process and transport antifungal humoral response, as seen in other organisms such as *L. monocytogenes* [28]. Elevation of humoral immunity was previously reported in larvae colonised with *Bacillus thuringiensis* and linked to elevated haemolymph phenoloxidase and lysozyme activity, and corresponding decreased coagulation index and phenoloxidase activity in the hemocytes [53]. However, further work is required to elucidate the mechanisms responsible for this reaction in the larvae during CDI.

## 5. Conclusions

This study was designed to further develop the use of *G. mellonella* larva as an in vivo model for CDI and phage therapy using different phage therapy regimens. This was done by linking expression profiles of *G. mellonella* stress genes to survival, colonisation and toxin levels in experimental insects during phage therapy. We found a strong relationship between two sets of data; the survival colonisation and toxin rates, and various *G. mellonella* stress-related genes. Hence, these genes served as molecular indicators to simulate symptoms monitoring in very refined in vivo models, the *G. mellonella* larva, and their application to study CDI and phage therapy.

## Figures and Tables

**Table 1:** Treatment regimens for the controls, prophylactic and remedial phage therapy used in this study.

Experimental Groups	Treatments	Time (h)				
		0	2	24	48	72
1	Control 1—Untreated	-	-	-	-	-
2	Control 2—Empty needle	N	N	-	-	-
3	Control 3—BHI	BHI	BHI	-	-	-
4	Control 4—Bacteria (CD105LC2)	B	BHI	-	-	-
5	Phage/bacteria co-infection	P+B	BHI	-	-	-
6	Remedial regimen	B	P	-	-	-
7	Prophylactic regimen	P	B	-	-	-
8	Control 5—Phage only	P	BHI	-	-	-

Insects were treated with either an empty needle (N), 10 µL of either brain heart infusion broth (BHI), 10^5^ CFU/larva of test bacterial strain CD105LC2 (B), 10^6^ PFU/larva of the 4-phage cocktail (P), a co-infection of the 4-phage cocktail and bacteria (P+B) or remained untreated (-) at the time points shown. Insects were culled at each relevant time point by incubating them at −20 °C for 20 min.

**Table 2:** Stress markers genes investigated during *C. difficile* infection and phage therapy regimens in *G. mellonella.*

Gene Function	Number of Genes	Gene Name/Locus Tag	Reference
Growth	9	GME-string_contig704.0, GME-string_contig233.0 Juvenile hormone epoxide hydrolase_1 Juvenile hormone epoxide hydrolase _2 Juvenile hormone binding_1 Juvenile hormone esterase Juvenile hormone inducible Juvenile hormone binding_3 Juvenile hormone binding_4	[28]
Cellular immunity	3	Contig19101_1 Contig20595_1 Contig15265_1	
Humoral immunity	1	Contig14128_1	
Infection	2	Moricin, Gloverin	
Reproduction	*2*	Ecdysteroid_regulated_pr Ecdysteroid_22-kinase	

**Table 3:** Average ELISA readings of *C. difficile* toxins from *G. mellonella* guts treated with phage cocktail.

Time (h)	Experimental Treatment Groups (OD_450_ Measurements)							
	1	2	3	4	5	6	7	8
	Untreated	Needle	BHI	CD105LC2	Co-Infection	Remedial	Prophylaxis	Phage
2	0.063	0.064	0.067	0.072	0.111	0.108	0.127	0.061
24	0.068	0.058	0.060	0.192	0.201	0.293	0.201	0.054
48	0.067	0.059	0.057	0.210	0.239	0.223	0.108	0.060
72	0.065	0.065	0.091	0.234	0.193	0.260	0.129	0.059

## References

[1] Olsen M.A., Young-Xu Y., Stwalley D., Kelly C.P., Gerding D.N., Saeed M.J., Mahé C., Dubberke E.R. (2016). The burden of *Clostridium difficile* infection: Estimates of the incidence of CDI from U.S. Administrative databases. BMC Infect. Dis..

[2] Kwon J.H., Olsen M.A., Dubberke E.R. (2015). The Morbidity, Mortality, and Costs Associated with *Clostridium difficile* Infection. Infect. Dis. Clin. N. Am..

[3] Bacci S., Molbak K., Kjeldsen M.K., Olsen K.E. (2011). Binary toxin and death after Clostridium difficile infection. Emerg. Infect. Dis..

[4] Lyerly D.M., Krivan H.C., Wilkins T.D. (1988). *Clostridium difficile*: Its disease and toxins. Clin. Microbiol. Rev..

[5] Legenza L., Barnett S., Rose W., Bianchini M., Safdar N., Coetzee R. (2018). Epidemiology and outcomes of *Clostridium difficile* infection among hospitalised patients: Results of a multicentre retrospective study in South Africa. BMJ Glob. Health.

[6] Lessa F.C., Mu Y., Bamberg W.M., Beldavs Z.G., Dumyati G.K., Dunn J.R., Farley M.M., Holzbauer S.M., Meek J.I., Phipps E.C. (2015). Burden of *Clostridium difficile* Infection in the United States. N. Engl. J. Med..

[7] Lessa F.C., Gould C.V., McDonald L.C. (2012). Current Status of *Clostridium difficile* Infection Epidemiology. Clin. Infect. Dis..

[8] Kuijper E.J., Barbut F., Brazier J.S., Kleinkauf N., Eckmanns T., Lambert M.L., Drudy D., Fitzpatrick F., Wiuff C., Brown D.J. (2008). Update of *Clostridium difficile* infection due to PCR ribotype 027 in Europe, 2008. Eurosurveillance.

[9] Akerlund T., Persson I., Unemo M., Noren T., Svenungsson B., Wullt M., Burman L.G. (2008). Increased sporulation rate of epidemic *Clostridium difficile* Type 027/NAP1. J. Clin. Microbiol..

[10] Vonberg R.P., Kuijper E.J., Wilcox M.H., Barbut F., Tull P., Gastmeier P., van den Broek P.J., Colville A., The European C. difficile-Infection Control Group, The European Centre for Disease Prevention and Control (ECDC) (2008). Infection control measures to limit the spread of *Clostridium difficile*. Clin. Microbiol. Infect..

[11] Peng Y.Z., Huang G.T. (2016). Phage therapy for bacterial infection of burn. Zhonghua Shao Shang Za Zhi.

[12] Shah D., Dang M.D., Hasbun R., Koo H.L., Jiang Z.D., DuPont H.L., Garey K.W. (2010). *Clostridium difficile* infection: Update on emerging antibiotic treatment options and antibiotic resistance. Expert Rev. Anti-Infect. Ther..

[13] Debast S.B., Bauer M.P., Kuijper E.J. (2014). European Society of Clinical Microbiology and Infectious Diseases: Update of the Treatment Guidance Document for *Clostridium difficile* Infection. Clin. Microbiol. Infect..

[14] Koss C.A., Baras D.C., Lane S.D., Aubry R., Marcus M., Markowitz L.E., Koumans E.H. (2012). Investigation of Metronidazole Use during Pregnancy and Adverse Birth Outcomes. Antimicrob. Agents Chemother..

[15] Nelson R.L., Suda K.J., Evans C.T. (2017). Antibiotic treatment for *Clostridium difficile*-associated diarrhoea in adults. Cochrane Database Syst. Rev..

[16] Baines S.D., Wilcox M.H. (2015). Antimicrobial Resistance and Reduced Susceptibility in *Clostridium difficile*: Potential Consequences for Induction, Treatment, and Recurrence of C. difficile Infection. Antibiotics.

[17] Zucca M., Scutera S., Savoia D. (2013). Novel avenues for *Clostridium difficile* infection drug discovery. Expert Opin. Drug Discov..

[18] Giau V.V., Lee H., An S.S.A., Hulme J. (2019). Recent advances in the treatment of *C. difficile* using biotherapeutic agents. Infect. Drug Resist..

[19] Slopek S., Weber-Dabrowska B., Dabrowski M., Kucharewicz-Krukowska A. (1987). Results of bacteriophage treatment of suppurative bacterial infections in the years 1981-1986. Arch. Immunol. Ther. Exp..

[20] Abedon S.T., Kuhl S.J., Blasdel B.G., Kutter E.M. (2011). Phage treatment of human infections. Bacteriophage.

[21] Brüssow H. (2012). What is needed for phage therapy to become a reality in Western medicine?. Virology.

[22] Loc-Carrillo C., Abedon S.T. (2011). Pros and cons of phage therapy. Bacteriophage.

[23] Hargreaves K.R., Clokie M.R.J. (2014). *Clostridium difficile* phages: Still difficult?. Front. Microbiol..

[24] Nale J.Y., Spencer J., Hargreaves K.R., Buckley A.M., Trzepiński P., Douce G.R., Clokie M.R.J. (2016). Bacteriophage Combinations Significantly Reduce *Clostridium difficile* Growth In Vitro and Proliferation In Vivo. Antimicrob. Agents Chemother..

[25] Nale J.Y., Chutia M., Carr P., Hickenbotham P., Clokie M.R.J. (2016). ‘Get in early’; biofilm and wax moth (*Galleria mellonella*) models reveal new insights into the therapeutic potential of *Clostridium difficile* bacteriophages. Front. Microbiol..

[26] Nale J., Redgwell T.A., Millard A., Clokie M.R.J. (2018). Efficacy of an Optimised Bacteriophage Cocktail to Clear *Clostridium difficile* in a Batch Fermentation Model. Antibiotics.

[27] Best E.L., Freeman J., Wilcox M.H. (2012). Models for the study of *Clostridium difficile* infection. Gut Microbes.

[28] Mukherjee K., Hain T., Fischer R., Chakraborty T., Vilcinskas A. (2013). Brain infection and activation of neuronal repair mechanisms by the human pathogen *Listeria monocytogenes* in the lepidopteran model host *Galleria mellonella*. Virulence.

[29] Mukherjee K., Altincicek B., Hain T., Domann E., Vilcinskas A., Chakraborty T. (2010). *Galleria mellonella* as a Model System for Studying Listeria Pathogenesis. Appl. Environ. Microbiol..

[30] Beeton M.L., Alves D.R., Enright M.C., Jenkins A.T.A. (2015). Assessing phage therapy against *Pseudomonas aeruginosa* using a *Galleria mellonella* infection model. Int. J. Antimicrob. Agents.

[31] Viegas S., Mil-Homens D., Fialho A., Arraiano C. (2013). The Virulence of *Salmonella enterica* Serovar Typhimurium in the Insect Model *Galleria mellonella* Is Impaired by Mutations in RNase E and RNase III. Appl. Environ. Microbiol..

[32] Vogel H., Altincicek B., Glöckner G., Vilcinskas A. (2011). A comprehensive transcriptome and immune-gene repertoire of the lepidopteran model host *Galleria mellonella*. BMC Genom..

[33] Hargreaves K.R., Kropinski A.M., Clokie M.R.J. (2014). What Does the Talking?: Quorum Sensing Signalling Genes Discovered in a Bacteriophage Genome. PLoS ONE.

[34] Weld R.J., Butts C., Heinemann J.A. (2004). Models of phage growth and their applicability to phage therapy. J. Theor. Biol..

[35] O‘Sullivan L., Buttimer C., McAuliffe O., Bolton D., Coffey A. (2016). Bacteriophage-based tools: Recent advances and novel applications. F1000Res.

[36] Shan J., Ramachandran A., Thanki A.M., Vukusic F.B.I., Barylski J., Clokie M.R.J. (2018). Bacteriophages are more virulent to bacteria with human cells than they are in bacterial culture; insights from HT-29 cells. Sci. Rep..

[37] Flecknell P. (2002). Replacement, reduction and refinement. Altex.

[38] Meyers J.R. (2018). Zebrafish: Development of a Vertebrate Model Organism. Curr. Protoc. Essent. Lab. Tech..

[39] Ramarao N., Nielsen-Leroux C., Lereclus D. (2012). The Insect *Galleria mellonella* as a Powerful Infection Model to Investigate Bacterial Pathogenesis. J. Vis. Exp..

[40] Mukherjee K., Abu Mraheil M., Silva S., Müller D., Cemic F., Hemberger J., Hain T., Vilcinskas A., Chakraborty T. (2011). Anti-Listeria activities of *Galleria mellonella* hemolymph proteins. Appl. Environ. Microbiol..

[41] Kay S., Edwards J., Brown J., Dixon R. (2019). *Galleria mellonella* Infection Model Identifies Both High and Low Lethality of *Clostridium perfringens* Toxigenic Strains and Their Response to Antimicrobials. Front. Microbiol..

[42] Ramesh V., Fralick J.A., Rolfe R.D. (1999). Prevention of *Clostridium difficile*-induced ileocecitis with Bacteriophage. Anaerobe.

[43] Govind R., Fralick J.A., Rolfe R.D. (2011). In vivo lysogenization of a *Clostridium difficile* bacteriophage ΦCD119. Anaerobe.

[44] Buckley A.M., Spencer J., Maclellan L.M., Candlish D., Irvine J.J., Douce G.R. (2013). Susceptibility of Hamsters to *Clostridium difficile* Isolates of Differing Toxinotype. PLoS ONE.

[45] Lange A., Schäfer A., Bender A., Steimle A., Beier S., Parusel R., Frick J.-S. (2018). *Galleria mellonella*: A Novel Invertebrate Model to Distinguish Intestinal Symbionts From Pathobionts. Front. Immunol..

[46] Coates C.J., Lim J., Harman K., Rowley A.F., Griffiths D.J., Emery H., Layton W. (2019). The insect, *Galleria mellonella*, is a compatible model for evaluating the toxicology of okadaic acid. Cell Biol. Toxicol..

[47] Chippendale G.M., Reddy G.P.V. (1974). Dietary carbohydrates: Role in feeding behaviour and growth of the Southwestern corn borer, *Diatraea grandiosella*. J. Insect Physiol..

[48] Ge L., Jiang Y., Xia T., Song Q., Stanley D., Kuai P., Lu X., Yang G., Wu J. (2015). Silencing a sugar transporter gene reduces growth and fecundity in the brown planthopper, *Nilaparvata lugens* (Stål) (Hemiptera: Delphacidae). Sci. Rep..

[49] Mukherjee K., Fischer R., Vilcinskas A. (2012). Histone acetylation mediates epigenetic regulation of transcriptional reprogramming in insects during metamorphosis, wounding and infection. Front. Zool..

[50] Farhadian S.F., Suárez-Fariñas M., Cho C.E., Pellegrino M., Vosshall L.B. (2012). Post-fasting olfactory, transcriptional, and feeding responses in *Drosophila*. Physiol. Behav..

[51] Browne N., Heelan M., Kavanagh K. (2013). An analysis of the structural and functional similarities of insect hemocytes and mammalian phagocytes. Virulence.

[52] Mowlds P., Kavanagh K. (2008). Effect of pre-incubation temperature on susceptibility of *Galleria mellonella* larvae to infection by *Candida albicans*. Mycopathologia.

[53] Grizanova E.V., Dubovskiy I.M., Whitten M.M.A., Glupov V.V. (2014). Contributions of cellular and humoral immunity of *Galleria mellonella* larvae in defence against oral infection by Bacillus thuringiensis. J. Invertebr. Pathol..

